# Sex differences in prevalence and characteristics of imaging-detected atherosclerosis: a population-based study

**DOI:** 10.1093/ehjci/jeae217

**Published:** 2024-08-19

**Authors:** Eva Swahn, Sofia Sederholm Lawesson, Joakim Alfredsson, Mats Fredrikson, Oskar Angerås, Olov Duvernoy, Gunnar Engström, Maria J Eriksson, Erika Fagman, Bengt Johansson, Linda Johnson, Nina Johnston, Johan Ljungberg, Maria Mannila, Maria Nordendahl, Jonas Oldgren, Elmir Omerovic, Ellen Ostenfeld, Margaretha Persson, Annika Rosengren, Linn Skoglund Larsson, Johan Sundström, Mia Söderberg, Carl Johan Östgren, Karin Leander, Tomas Jernberg

**Affiliations:** Department of Cardiology, Linköping University, SE-58185 Linköping, Sweden; Department of Health, Medicine and Caring Sciences, Linköping University, SE-58183 Linköping, Sweden; Department of Cardiology, Linköping University, SE-58185 Linköping, Sweden; Department of Health, Medicine and Caring Sciences, Linköping University, SE-58183 Linköping, Sweden; Department of Cardiology, Linköping University, SE-58185 Linköping, Sweden; Department of Health, Medicine and Caring Sciences, Linköping University, SE-58183 Linköping, Sweden; Forum Östergötland, Faculty of Medicine and Health Sciences, Linköping University, Linköping, Sweden; Inflammation and Infection, Department of Experimental and Clinical Medicine, Faculty of Medicine and Health Sciences, Linköping University, Linköping, Sweden; Department of Cardiology, Sahlgrenska University Hospital, Gothenburg, Sweden; Department of Molecular and Clinical Medicine, Sahlgrenska Academy, University of Gothenburg, Gothenburg, Sweden; Department of Surgical Sciences, Section of Radiology, Uppsala University, Uppsala, Sweden; Department of Clinical Sciences in Malmö, Lund University, Malmö, Sweden; Department of Molecular Medicine and Surgery, Karolinska Institute, Stockholm, Sweden; Department of Clinical Physiology, Karolinska University Hospital, Stockholm, Sweden; Department of Radiology, Sahlgrenska University Hospital, Gothenburg, Sweden; Department of Radiology, Institute of Clinical Sciences, Sahlgrenska Academy, University of Gothenburg, Gothenburg, Sweden; Department of Public Health and Clinical Medicine, Umeå University, Umeå, Sweden; Department of Clinical Sciences in Malmö, Lund University, Malmö, Sweden; Department of Medical Sciences, Cardiology, Uppsala University, Uppsala, Sweden; Department of Public Health and Clinical Medicine, Medicine and Heart Centre, Umeå University, Umeå, Sweden; Department of Cardiology and Clinical Genetics, Karolinska University Hospital, Stockholm, Sweden; Department of Public Health and Clinical Medicine, Family Medicine, Umeå University, Umeå, Sweden; Department of Medical Sciences, Cardiology, Uppsala University, Uppsala, Sweden; Uppsala Clinical Research Center, Uppsala University, Uppsala, Sweden; Department of Cardiology, Sahlgrenska University Hospital, Gothenburg, Sweden; Department of Molecular and Clinical Medicine, Sahlgrenska Academy, University of Gothenburg, Gothenburg, Sweden; Department of Clinical Sciences Lund, Clinical Physiology, Lund University, Skåne University Hospital, Lund, Sweden; Department of Clinical Sciences in Malmö, Lund University, Malmö, Sweden; Department of Internal Medicine, Skåne University Hospital, Malmö, Sweden; Department of Molecular and Clinical Medicine, Sahlgrenska Academy, University of Gothenburg, Gothenburg, Sweden; Department of Medicine Geriatrics and Emergency Medicine, Sahlgrenska University Hospital Östra Hospital, Gothenburg, Sweden; Department of Public Health and Clinical Medicine, Umeå University, Umeå, Sweden; Department of Medical Sciences, Uppsala University, Uppsala, Sweden; The George Institute for Global Health, University of New South Wales, Sydney, Australia; Occupational and Environmental Medicine, School of Public Health and Community Medicine, Institute of Medicine, Sahlgrenska Academy, University of Gothenburg, Gothenburg, Sweden; CMIV Centre of Medical Image Science and Visualization, Linköping University, Linköping, Sweden; Department of Health, Medicine and Caring Sciences, Linköping University, Linköping Sweden; Unit of Cardiovascular and Nutritional Epidemiology, Institute of Environmental Medicine, Karolinska Institutet, Stockholm, Sweden; Department of Clinical Sciences, Danderyd University Hospital, Karolinska Institutet, Stockholm, Sweden

**Keywords:** sex characteristics, coronary computed tomography angiography, atherosclerosis, coronary artery disease, carotid artery disease, ultrasonography

## Abstract

**Aims:**

Men are more likely to suffer a myocardial infarction than women, but population-based studies on sex differences in imaging-detected atherosclerosis are lacking. The aims were to assess sex differences in the prevalence of imaging-detected coronary and carotid atherosclerosis, as well as multivariable adjusted associations between sex and atherosclerosis.

**Methods and results:**

Participants aged 50–65, recruited from the general population to the Swedish Cardiopulmonary bioImage Study (SCAPIS), were included in this population-based cross-sectional study. Comprehensive diagnostics, including coronary computed tomography angiography and carotid ultrasound, were performed. The image findings were any coronary atherosclerosis, coronary stenosis ≥ 50%, segment involvement score (SIS) ≥ 4, coronary artery calcium score (CACS) > 100, and any ultrasound-detected carotid plaque. In 25 580 participants (50% women), men had more hypertension (20.3% vs. 17.0%), hyperlipidaemia (9.0% vs. 5.5%), and diabetes (8.5% vs. 4.7%). The prevalence was 56.2% vs. 29.5% for any coronary atherosclerosis (*P* < 0.01), 9.0% vs. 2.3% for coronary stenosis ≥ 50% (*P* < 0.01), 20.2% vs. 5.3% for SIS ≥ 4 (*P* < 0.01), 18.2% vs. 5.6% for CACS > 100 (*P* < 0.01), and 60.9% vs. 48.7% for carotid plaque (*P* < 0.01), in men vs. women, respectively. Multivariable adjustment only marginally changed these associations: odds ratios (ORs) (95% confidence interval): 2.75 (2.53–2.99) for coronary atherosclerosis, 2.88 (2.40–3.45) for coronary stenosis ≥ 50%, 3.99 (3.50–4.55) for SIS ≥ 4, 3.29 (2.88–3.75) for CACS > 100, and 1.57 (1.45–1.70) for carotid plaque.

**Conclusion:**

Men had higher prevalence of imaging-detected carotid and coronary atherosclerosis with prevalence in women aged 65 corresponding to men 11–13 years younger. The associations remained after extensive multivariable adjustment.

## Graphical Abstract

**Structured Graphical Abstract:**
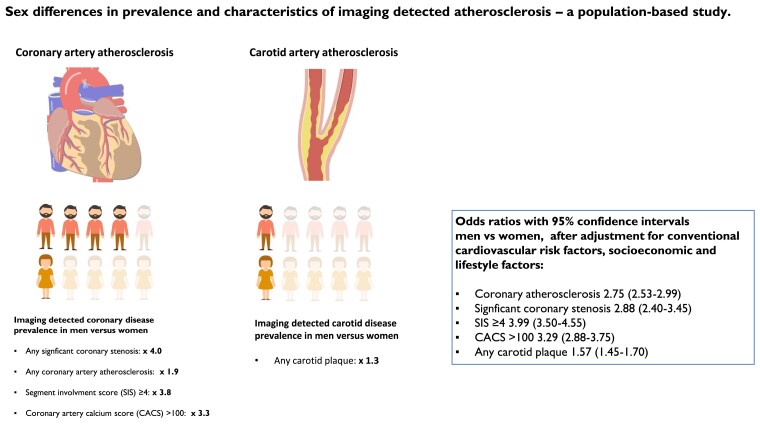
Prevalence of imaging-detected atherosclerosis in the general Swedish population, aged 50–65 years.

**See the editorial comment for this article ‘Imaging detected coronary and carotid atherosclerosis in the general population: is prevalence still different between sexes?’, by R. Liga *et al.*, https://doi.org/10.1093/ehjci/jeae218.**

## Introduction

Atherosclerotic cardiovascular disease (ASCVD) is the leading cause of death in both sexes, with women affected on average 5–7 years later than men.^1^ The risk factors associated with ASCVD are similar in men and women, although smoking, diabetes, and hypertension may have a stronger relative impact in women.^2,3^ In addition, there is accumulating evidence for sex-specific risk factors, particularly reproductive-related risk factors in women.^4^ Prospective cohort studies have shown that men, compared with women, have approximately twice the lifetime risk of incident myocardial infarction (MI), even when adjusting for conventional ASCVD risk factors,^5^ which is consistent with sex differences previously shown in coronary calcification.^6,7^ However, direct visualization methods, such as coronary computed tomography angiography (CCTA), are needed to study total plaque burden, including non-calcified components and degree of stenosis. In the first prevalence report of CCTA-detected coronary artery atherosclerosis and its association with coronary artery calcium scores (CACSs) from the Swedish Cardiopulmonary bioImage Study (SCAPIS) study, the aim was to report from the whole population included. In that report, all subjects with previous cardiovascular disease were excluded.^8^ A simple gender comparison was done without any deeper analysis of the burden of atherosclerosis in different arterial beds. Thus, to date, there is no large study in a randomly selected sample of the general population focusing on sex differences in prevalence, distribution, and characteristics of imaging-detected coronary and carotid atherosclerosis. In addition, the relative importance of lifestyle, socioeconomic, and conventional cardiovascular risk factors for the development of atherosclerosis in different locations in men and women needs to be assessed in a population-based sample. In terms of female-specific cardiovascular risk factors, we reported last year from the SCAPIS study on associations between subclinical coronary artery disease (CAD) assessed by CCTA and a history of adverse pregnancy outcomes.^4^

The aims of this study were to examine sex differences in prevalence and characteristics of imaging-detected coronary and carotid atherosclerosis in middle age and whether an association between sex and atherosclerosis in coronary and carotid arteries can be explained by differences in lifestyle, socioeconomic, and conventional risk factors. An additional aim was to assess the relative importance of risk factors for the presence of coronary and carotid atherosclerosis in men and women.

## Methods

**Figure 1:**
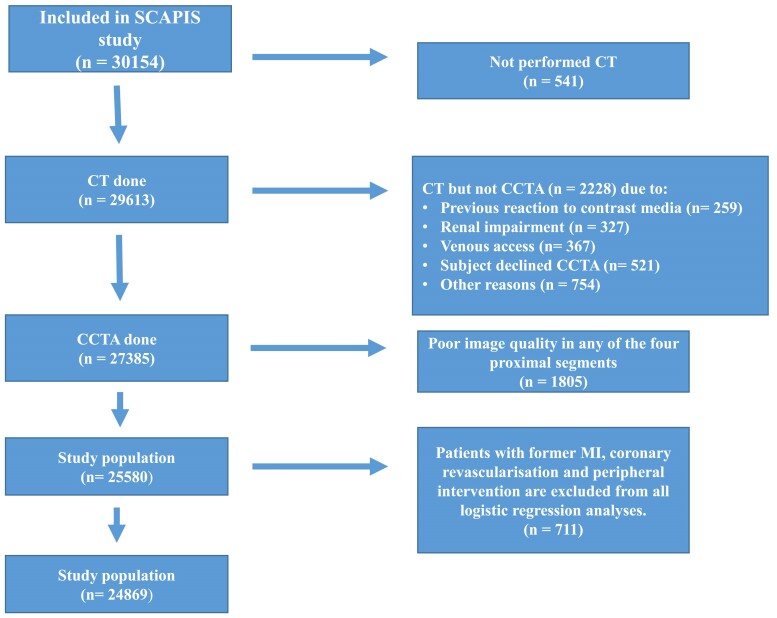
Inclusion flowchart.

The SCAPIS is a population-based, cross-sectional study described in detail elsewhere, including a specific description of the cardiac imaging performed^8^ (see Supplementary data online, *Appendix S1*). In short, 30 154 randomly invited individuals aged 50–65 (50.3% of all initially invited) were recruited at six sites from the Swedish census registry between 2013 and 2018. After giving informed consent, all invited individuals underwent cardiovascular examinations, including ultrasound of the carotid arteries and CCTA with a dual-source CT scanner equipped with a Stellar Detector (Somatom Definition Flash, Siemens Medical Solutions). In addition, anthropometric data, blood pressure (BP), and laboratory data were collected, as well as questionnaire data on perceived health, medication, socioeconomic situation, and education. For the interpretation of CCTA imaging, the 18 coronary segment model was used, where segments 1–3, 5–7, 9, 11–13, and 17 were compulsory to report. Women and men undergoing CCTA with interpretable images of the proximal segments (*n* = 25 580) constituted our study population (*Figure 1*). Sex was defined according to the individual’s Swedish personal identity number. Gender was not considered in the design of the study.

### Outcome measures

In the current study, the following coronary and carotid image findings were used: (i) coronary atherosclerosis specified as the presence of any CCTA-detected coronary atherosclerosis; (ii) the presence of any CCTA-detected coronary stenosis ≥ 50%; (iii) number of CCTA-detected coronary segments affected by atherosclerosis [segment involvement score (SIS)] dichotomized as <or ≥4; (iv) CACS dichotomized to ≤100 or >100 Agatston units; and (v) carotid atherosclerosis defined as any ultrasound-detected plaque in the carotid arteries.

### Ethics

The multicentre study was approved by the ethics review board in Umeå (number 2010-228-31M) and complied with the Declaration of Helsinki. Patient data were pseudonymized to protect the identities of individuals. The participants gave written informed consent.

### Statistical analyses

Continuous variables are presented as mean and standard deviation or median and interquartile range as appropriate. Categorical variables are presented as counts and percentages. Comparisons between groups were performed by *χ*^2^ tests for categorical variables, Student’s *t*-test for normally distributed continuous variables and the Mann–Whitney *U* test for non-normally distributed continuous variables.

To examine sex differences in annual image findings rates, interactions between age and sex for all image outcomes were assessed with logistic regression analyses, including sex, age per year of increase, and the product between age and sex as an interaction term in the model.

To assess associations between sex and the five coronary and carotid image outcomes, logistic regression analyses were applied, using three different models. Results are presented as age and multivariable adjusted odds ratios (ORs) with 95% confidence intervals (CIs). In these analyses, study participants with previous MI, stroke, coronary revascularization, and intervention of peripheral artery disease were excluded. In Model 1, only sex and age were incorporated. In Model 2, the following socioeconomic and lifestyle variables were added to Model 1 (for definitions, see the Supplementary data online): alcohol consumption, sedentary percentage of accelerometer wear time, moderate and vigorous intensity physical activity, percentage of accelerometer wear time, smoking, level of education, financial strain, foreign-born, single household, and suspected sleep apnoea. In Model 3, the following conventional ASCVD risk factors were added to Model 2: body mass index (BMI), waist–hip ratio, systolic BP, pharmacologically treated hypertension, pharmacologically treated hyperlipidaemia, diabetes mellitus, heredity for MI, C-reactive protein, total cholesterol, high-density lipoprotein cholesterol, and low-density lipoprotein cholesterol.

To assess the relative importance of risk factors in men and women, sex-specific logistic regression analyses were performed, with results presented as crude and multivariable adjusted OR—the latter including the same covariates as in Model 3, as previously described. In addition, using the total cohort, logistic regression analyses were performed to assess whether there were interactions between any factor and sex regarding the five imaging outcome measures. This was done by including interaction terms (sex times the specific factor) into the models, including sex and the factor of interest, as well as into multivariable models, including the same covariates as in Model 3, as previously described.

In all analyses, due to multiple comparisons, a *P*-value of <0.01 was considered statistically significant. All statistical analyses were performed using IBM SPSS Statistics 28 (IBM, Armonk, NY, USA).

## Results

### Baseline characteristics

Of 25 580 individuals included in the study, 12 755 (50%) were men and 12 825 (50%) women, with a mean age of 57.4 in both sexes. Smoking rates were similar in men and women (12.4% and 12.8%), while men had higher BMI (27.3 vs. 26.3) and higher systolic BP (128.7 vs. 123.0 mmHg). Higher education was more frequent in women, who also reported higher stress levels than men. Regarding clinical characteristics, men were more likely to have hypertension (20.3% vs. 17.0%), hyperlipidaemia (9.0% vs. 5.5%), previous MI (1.8% vs. 0.5%), stroke (1.6% vs. 1.1%), coronary revascularization (1.1% vs. 0.2%), and diabetes than women (8.5% vs. 4.7%) (*Table 1*).

**Table 1:** Characteristics of study participants

	Total *n* = 25580	Men *n* = 12755	Women *n* = 12825	*p*
**Sociodemographic data**				
Age, mean (SD) years	57.4 (4.3)	57.4 (4.4)	57.4 (4.3)	0.20
Married	18548 (74.4)	9678 (78.2)	8870 (70.7)	<0.01
Living alone	4652(18.6)	2048(16.5)	2604(20.7)	<0.01
Foreign born	3828 (15.3)	1863 (15.0)	1965 (15.6)	0.20
**Anthropometry**				
BMI, mean (SD) kg/m^2^	26.8 (4.3)	27.3 (3.8)	26.3 (4.6)	<0.01
Waist, mean (SD) cm	93.9 (12.6)	99.3 (10.9)	88.9 (12.1)	<0.01
Waist-hip-ratio, mean (SD)	0.92 (0.09)	0.97 (0.07)	0.86 (0.07)	<0.01
**Smoking and alcohol habits**				
Current smoker	3108 (12.6)	1520 (12.4)	1588 (12.8)	<0.01
Former smoker	8961 (36.2)	4122 (33.5)	4839 (38.9)	
Packyears, median (IQR)	12.0 (5.0-22.5)	12.9 (5.3-25.0)	11.9 (5.0-21.0)	<0.01
Never using alcohol	2025 (8.1)	880 (7.1)	1145 (9.1)	<0.01
Problematic alcohol use	4598 (18.0)	3438 (27.0)	1160 (9.0)	<0.01
**Socioeconomic data**				
Higher education	11366(45.5)	5106 (41.2)	6260 (49.8)	<0.01
Employed	21222 (85.2)	10646 (86.1)	10576 (54.3)	<0.01
Difficulties managing regular expenses, last 12 months	1284 (5.2)	609 (4.9)	675 (5.4)	0.11
Ability to find SEK 20 000 in a week for unforeseen events	22846 (93.2)	11481 (94.4)	11365 (92.1)	<0.01
Perception of constant stress last year	5165 (20.9)	1987 (16.2)	3178 (25.6)	<0.01
**Physical activity**				
Moderate-and vigorous intensity PA^a^, median (IQR)	6 (4-8)	6 (4-8)	6 (4-8)	<0.01
Being sedentary^a^, median (IQR)	54 (47-61)	56 (49-63)	52 (45-59)	<0.01
**Co-morbidity**				
Medically treated hypertension	4618 (18.7)	2492 (20.3)	2126 (17.0)	<0.01
Medically treated hyperlipidemia	1795 (7.3)	1108 (9.0)	687 (5.5)	<0.01
Diabetes	1681 (6.6)	1079 (8.5)	602 (4.7)	<0.01
Previous MI	288 (1.2)	221 (1.8)	67 (0.5)	<0.01
Previous revascularization	158 (0.6)	132 (1.1)	26 (0.2)	<0.01
Previous stroke	326 (1.3)	192 (1.6)	134 (1.1)	<0.01
Suspected sleep apnea	1963 (7.7)	1385 (10.9)	578 (4.5)	<0.01
**Heredity**				
Heredity for MI	1674 (6.7)	764 (6.2)	910 (7.2)	<0.01
**Blood pressure and laboratory data**				
Systolic blood pressure, mean (SD) mmHg	125.8 (16.8)	128.7 (15.5)	123.0 (17.6)	<0.01
Diastolic blood pressure, mean (SD) mmHg	77.5 (10.5)	78.4 (10.1)	76.6 (10.7)	<0.01
LDL cholesterol, mean (SD) mmol/L	3.5 (0.9)	3.5 (1.0)	3.4 (1.0)	0.74
HDL cholesterol, mean (SD) mmol/L	1.6 (0.5)	1.4 (0.4)	1.8 (0.5)	<0.01
HbA1c,mean (SD) mmol/mL	36.2 (5.9)	36.5 (6.6)	36.1 (5.2)	<0.01
C Reactive Protein, median (IQR) mg/L	1.0 (0.6-2.1)	1.0 (0.6-2.0)	1.0 (0.6-2.2)	0.10
eGFR, mean (SD) ml/min/1.73 m^2^	84.7 (14.3)	86.4 (14.0)	83.0 (14.4)	<0.01

Values are presented as *n* (%) unless otherwise indicated.

BMI, body mass index; COPD, chronic obstructive pulmonary disease; CPM, mean vector magnitude in counts per minute; eGFR, estimated glomerular filtration rate; HbA1C, glycosylated haemoglobin type A1; HDL, high-density lipoprotein; IQR, interquartile range; LDL, low-density lipoprotein; MI, myocardial infarction; PA, physical activity; PAD; peripheral artery disease; SD, standard deviation.

^a^Percentage of accelerometer wear time.

### Prevalence and characteristics of atherosclerosis in coronary and carotid arteries

Men had consistently more coronary atherosclerosis than women: any coronary atherosclerosis in 56.2% vs. 29.5%, *P* < 0.01; coronary stenosis ≥ 50% in 9.1% vs. 2.3%, *P* < 0.01; SIS ≥ 4 in 20.2% vs. 5.3%, *P* < 0.01; and CACS > 100 in 18.2% vs. 5.6%, *P* < 0.01. CACS > 0 was found in 27.4% of women and 53.7% of men. Among those with coronary atherosclerosis, men had more calcified (95.3% vs. 92.2%, *P* < 0.01) and non-calcified (20.8% vs. 18.1%, *P* < 0.01) plaques than women. Carotid plaques were present in 60.9% of men and 48.7% of women, *P* < 0.01 (*Table 2*).

**Table 2:** Prevalence of atherosclerosis

	Total	Men	Women	*P*
**CCTA**	***n* = 25 580**	***n* = 12 755**	***n* = 12 825**	
Any coronary atherosclerosis	10 952 (42.8)	7164 (56.2)	3788 (29.5)	<0.01
Stenosis ≥ 50%	1458 (5.7)	1159 (9.1)	299 (2.3)	<0.01
One vessel disease	1114 (4.4)	875 (6.9)	239 (1.9)	<0.01
Two vessel disease	229 (0.9)	187 (1.5)	42 (0.3)	<0.01
Three vessel disease	78 (0.3)	69 (0.5)	9 (0.1)	<0.01
Left main disease	48 (0.2)	38 (0.3)	10 (0.1)	<0.01
Proximal LAD disease	496 (1.9)	393 (3.1)	103 (0.8)	<0.01
Left main, proximal LAD or 3VD	526 (2.1)	420 (3.3)	106 (0.8)	<0.01
Segment involvement score ≥ 4	3254 (12.7)	2574 (20.2)	680 (5.3)	<0.01
**CACS**	***n* = 25181**	***n* = 12463**	***n* = 12718**	
CACS,^a^ median (IQR)	36 (8;129)	45 (10;162)	24 (6;79)	<0.01
CACS 0	15 008 (59.6)	5775 (46.3)	9233 (72.6)	<0.01
CACS 1–10	2878 (11.4)	1699 (13.6)	1179 (9.3)	
CACS 11–100	4311 (17.1)	2722 (21.8)	1589 (12.5)	
CACS 101–400	2047 (8.1)	1496 (12.0)	551 (4.3)	
CACS >400	937 (3.7)	771 (6.2)	166 (1.3)	
**Characteristics in those with atherosclerosis**	***n* = 10 952**	***n* = 7164**	***n* = 3788**	
Any calcified plaque	10 320 (94.2)	6828 (95.3)	3492 (92.2)	<0.01
Only calcified plaques	8758 (80.0)	5663 (79.0)	3095 (81.7)	<0.01
Any calcified stenosis ≥ 50%	1223 (11.2)	998 (13.9)	225 (5.9)	<0.01
Any non-calcified plaque	2173 (19.8)	1488 (20.8)	685 (18.1)	<0.01
Only non-calcified plaques	126 (1.2)	73 (1.0)	53 (1.4)	0.08
Any non-calcified stenosis ≥ 50%	356 (3.3)	264 (3.7)	92 (2.4)	<0.01
Ratio between CACS/SIS (median, IQR) *n* = 9806	18.0 (6.0–43.2)	20 (6.0–47.7)	15.0 (5.0–35.1)	<0.01
**Carotid atherosclerosis**	***n* = 25 580**	***n* = 12 755**	***n* = 12 825**	
Any carotid plaque	14007 (54.8)	7763 (60.9)	6244 (48.7)	<0.01
Bilateral carotid plaques	6368 (24.9)	3858 (30.2)	2510 (19.6)	<0.01

Values are presented as *n* (%) unless otherwise indicated.

3VD, three vessel disease; CACS, coronary artery calcium score; CAD, coronary atherosclerosis; CCTA, computed coronary tomography angiography; IQR, interquartile range; LAD, left anterior descending artery; SIS, segment involvement score.

^a^In study participants with CACS and SIS > 0.

**Figure 2:**
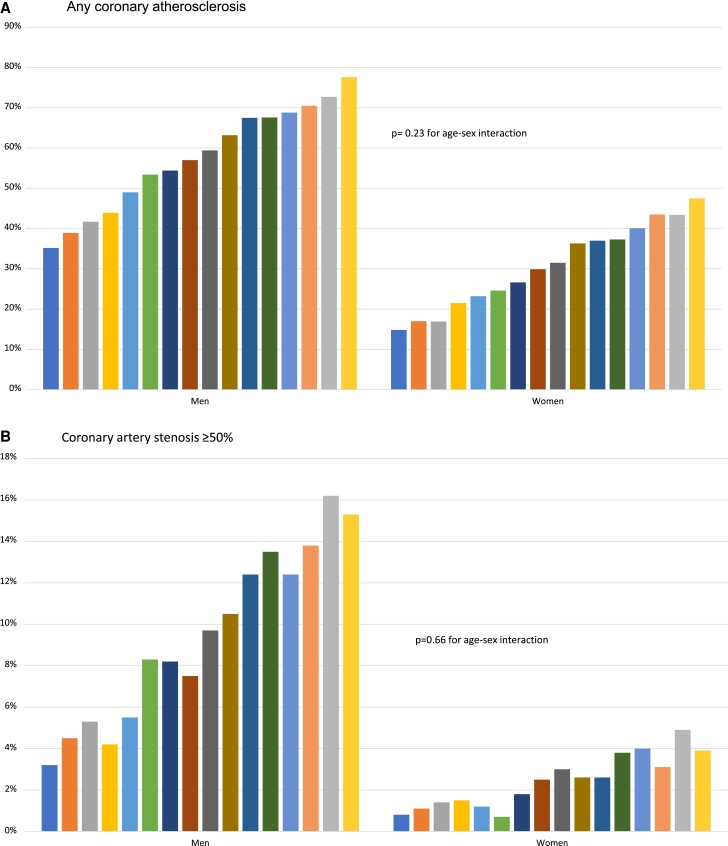

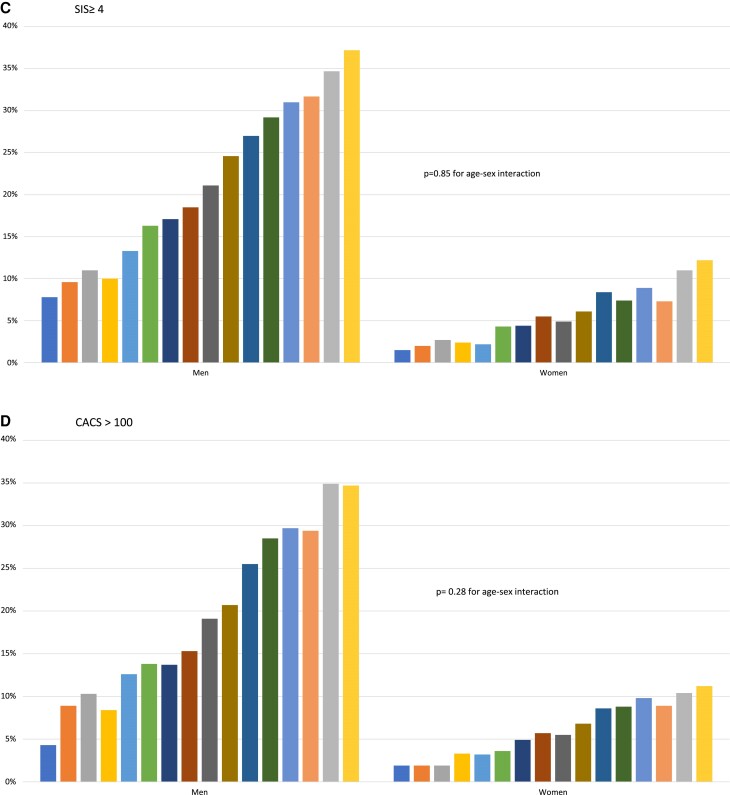

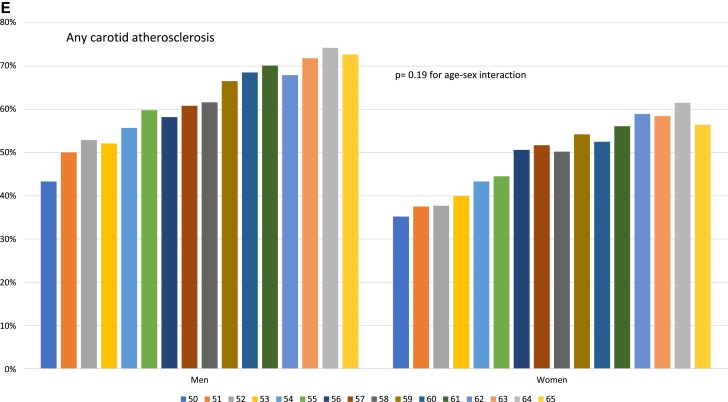
(*A*) Any coronary atherosclerosis in age groups per year of increase in men and women. (*B*) Coronary artery stenosis ≥ 50% in age groups per year of increase in men and women. (*C*) SIS ≥ 4 in age groups per year of increase in men and women. (*D*) CACS > 100 in age groups per year of increase in men and women. (*E*) Any carotid atherosclerosis in age groups per year of increase in men and women.

In all ages, atherosclerosis was more common in men than in women, with no significant difference in increased prevalence of any of the atherosclerosis image findings per year of life. The highest prevalence of atherosclerosis in women occurred in the oldest age strata, with women aged 65 years corresponding to men 11–13 years younger (*Figure 2*).

### Association between sex and imaging-detected atherosclerosis

**Figure 3:**
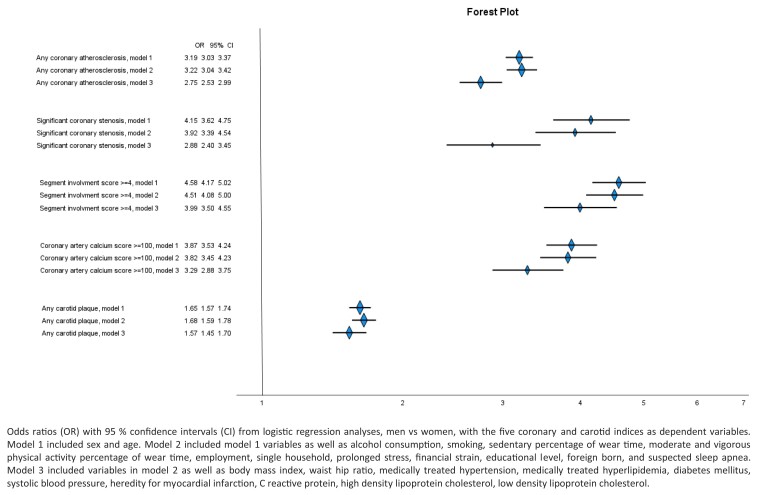
Risk of atherosclerosis in carotids or coronary arteries in middle-aged women and men.

After adjustment for age, the odds for all image findings were higher in men compared with women: any coronary atherosclerosis, OR 3.19 (95% CI 3.03–3.36); any coronary stenosis ≥ 50%, 4.15 (95% CI 3.62–4.76); SIS ≥ 4, OR 4.58 (95% CI 4.17–5.03); CACS >100, OR 3.87 (95% CI 3.53–4.24); and carotid plaque, OR 1.65 (95% CI 1.57–1.73) in men vs. women, respectively. Adding socioeconomic and lifestyle variables and suspected sleep apnoea (Model 2) did not substantially change the OR of atherosclerosis associated with male sex: OR 3.22 (95% CI 3.04–3.42) for any coronary atherosclerosis; 3.92 (95% CI 3.39–4.54) for any coronary stenosis ≥ 50%; 4.51 (95% CI 4.08–5.00) for SIS ≥ 4; 3.82 (95% CI 3.45–4.16) for CACS > 100; and 1.68 (95% CI 1.59–1.78) for any carotid plaque in men vs. women, respectively. After adding conventional cardiovascular risk factors (Model 3), the ORs were somewhat attenuated: 2.75 (95% CI 2.53–2.99); 2.88 (95% CI 2.40–3.45); 3.99 (95% CI 3.50–4.55); 3.29 (95% CI 2.88–3.75); and 1.57 (95% CI 1.45–1.70) for any coronary atherosclerosis, any coronary stenosis > 50%, SIS ≥ 4, CACS > 100, and any carotid plaque, in men vs. women, respectively (*Figure 3*).

### Associations between risk factors and atherosclerosis in women and men

In the fully adjusted model (Model 3), there were interactions between sex and smoking for any coronary atherosclerosis, with higher OR in women (1.53, 95% CI 1.40–1.68 vs. 1.39, 95% CI 1.21–1.59, *P* for interaction < 0.01), between sexes and being foreign born, with higher OR for men (1.30, 95% CI 1.15–1.46 vs. 1.02, 95% CI 0.91–1.15, *P* for interaction < 0.01). For SIS ≥ 4, the same interactions were found, with higher OR in women for smoking (3.45, 95% CI 2.69–4.50 vs. 1.30, 95% CI 1.17–1.46, *P* for interaction < 0.01) and higher OR in men for foreign-born (1.42, 95% CI 1.23–1.64 vs. 0.92, 95% CI 0.72–1.18, *P* for interaction < 0.01). For coronary stenosis ≥ 50% and CACS > 100, OR for smoking was higher in women (2.34, 95% CI 1.62–3.37 vs. 1.46, 95% CI 1.17–1.82, *P* for interaction = 0.02, and 3.12, 95% CI 2.45–3.99 vs. 1.49, 95% CI 1.26–1.76, *P* for interaction < 0.01). For coronary stenosis ≥ 50% and carotid disease, no interactions were found (see the Supplementary data online).

## Discussion

The SCAPIS study is the first study examining a general population of middle-aged men and women using CCTA and carotid ultrasound to assess the presence of coronary and carotid atherosclerosis. We found men to be 1.9 times more likely to have coronary atherosclerosis, 4.0 times more likely to have coronary obstruction, and 1.3 times more likely to have carotid atherosclerosis. The degree of imaging detected that atherosclerosis—measured as any coronary atherosclerosis, significant stenosis in any coronary artery, coronary segment involvement, calcium load, or carotid plaques—increased with age in both sexes but was substantially more prevalent in men in all age strata. Atherosclerosis prevalence in women aged 65 was similar to men 11–13 years younger. The higher relative sex difference in coronary atherosclerosis compared with carotid atherosclerosis is congruent with the higher relative risk in men vs. women in MI as compared with ischaemic stroke.^9^

After extensive adjustment for lifestyle and well-known major cardiovascular risk factors, such as socioeconomic status, smoking, physical activity, BMI, hypertension, hyperlipidaemia, diabetes mellitus, and family history, the associations between sex and atherosclerosis were only marginally affected, indicating that other factors are causal for these differences. This is in keeping with a study by Yoon *et al*.,^10^ who found almost unchanged associations between sex and atherosclerosis in adjusted models in a health screening programme that included CCTA data. Our findings are also in line with studies examining the association between sex and clinical events. In the Tromsø study, even after quite extensive adjustments, the incidence rate of MI was still twice as high in men as in women.^5^

In our study, men had more hypertension, hyperlipidaemia, and diabetes and were more sedentary, whereas women more often lived alone, had a higher level of education, and experienced more stress, which is in agreement with previous studies on the general population.^11^ In both men and women, the conventional cardiovascular risk factors age, smoking, hyperlipidaemia, hypertension, and diabetes were associated with all coronary and carotid atherosclerotic indices. These findings are congruent with results on incident MI from the INTERHEART study,^3^ the Copenhagen City Heart Study, and the Tromsø study.^5,12^

When comparing the relative importance of each risk factor, the association between smoking and coronary atherosclerosis was stronger in women. The more deleterious effect of smoking in women compared with men has been suggested previously, but the mechanisms are not fully understood. Potential explanations include higher absorption rates or more detrimental effects of toxins in women.^13^ Being born outside of Sweden was associated with coronary atherosclerosis in men only. Sweden has undergone vast demographic changes, particularly due to the increasing refugee influx in recent decades (SCB, Statistics Sweden), and it has been shown that the risk factor burden is higher in immigrants than in native Swedes.^14,15^ We can only speculate that there may be sex differences in lifestyle among these immigrant groups and perhaps increased psychosocial stress in men due to cultural expectations of the man as a family provider.

The relative early protection from coronary and carotid atherosclerosis in women needs to be investigated from another angle, as neither conventional risk factors nor socioeconomic factors seem to explain the sex differences. After menopausal transition and the sharp decline in oestrogen levels, the incidence of CAD in women rises.^16–18^ The role of oestrogen is supported by the fact that men with a disruptive oestrogen receptor mutation have a predisposition for early coronary atherosclerosis.^19^ The role of androgens in CAD development has been less studied than the potentially protective role of oestrogen. In the Multi-Ethnic Study of Atherosclerosis study,^20^ elevated free testosterone levels were associated with a greater CAC progression in women, and women with the androgenic polycystic ovary syndrome had higher Agatston scores compared with controls.^21^ There are still scarce data on how sex intervenes in the atherosclerosis process, with few mechanistic studies comparing atherosclerosis development between the sexes and non-coherent data from animal studies.^22,23^

### Limitations and strengths

This is the first population-based study using equal gender samples and extensive information on potential confounders and mediators to study sex differences in imaging-detected coronary and carotid atherosclerosis in the general middle-aged population. As such, the data can be considered high quality.

However, our findings need to be considered in light of certain limitations. First, as this is a cross-sectional study, we do not know when the different covariates we considered appeared in relation to the development of atherosclerosis, and conclusions about the extent to which these contribute to an increased risk of ASCVD linked to male sex should be interpreted with caution. Second, as the population included only men and women between 50 and 65 years, the results are not generalizable to other age groups. Third, in this study, we did not cover female-specific cardiovascular risk factors but have published a report from the SCAPIS study on this issue, as mentioned in the Introduction. Fourth, we did not have access to >70% coronary stenosis prevalence or plaque characteristics.

## Conclusion

In the general population aged 50–65 years, men had two to four times more coronary atherosclerosis and 1.3 times more carotid atherosclerosis, reflecting sex differences in acute coronary syndromes, which are greater than for ischaemic stroke. The associations between male sex and coronary and carotid atherosclerosis were only marginally attenuated when adjusting for important socioeconomic, lifestyle, and traditional cardiovascular risk factors, indicating that the higher risk of atherosclerosis in men can only partly be explained by sex differences in relation to these factors. Future research focusing on the mechanisms behind sex as a biological variable in atherosclerosis development is critical in order to mitigate the higher risk of ASCVD in men and to better prevent ASCVD in both sexes. In addition, imaging-detected atherosclerosis reflects a risk of subsequent cardiovascular events and implies the need for intensified preventive efforts. CCTA enables early intervention with a potential to prevent cardiovascular events later in life.

## Supplementary Material

jeae217_Supplementary_Data

## Data Availability

The data underlying this article will be shared on reasonable request to the corresponding author.
