# Supplementary material for: Sex differences in prevalence and characteristics of imaging-detected atherosclerosis: a population-based study
Source: Eur Heart J Cardiovasc Imaging. 2024 Aug 19;25(12):1663–72. doi: 10.1093/ehjci/jeae217 (PMC11601724; doi:10.1093/ehjci/jeae217)
Supplement: jeae217_Supplementary_Data [file jeae217_supplementary_data.pdf]

Online-Only Supplement

Sex differences in prevalence and characteristics of imaging detected atherosclerosis – a population-based study

Eva Swahn, Sofia Sederholm Lawesson et al.

sMethods

Coronary Computed Tomography Angiography (CCTA) and coronary artery calcium scoring (CACS)

For **CACS**, non-contrast images were obtained using ECG-gated CT imaging at 120 kV, and software was used for coronary artery calcium scoring (Volume Wizard; Siemens). Lesions >130 Hounsfield units in ≥3 adjacent pixels 1mm^3 were identified, and coronary artery calcium score expressed in Agatston Units computed as the sum of the products of each pixel calcification and an intensity factor.

Before **CCTA**, sublingual glyceryl nitrate was given to dilate the coronaries and in the case of high pulse beta-blockers were given to aim at a heart rate of 60 beats per minute. Thirty-six thoracic radiologists or cardiologists interpreted the images and entered the data into the database electronically. The images were reconstructed and visually scored for atherosclerosis using syngo.via software. For coronary artery disease assessment, defined by Society of Cardiovascular Computed Tomography, the eighteen coronary segment model was used, with the most clinically relevant segments (segments 1–3, 5–7, 9, 11–13, and 17) compulsory to report regarding presence and type of plaques.

Any coronary atherosclerosis was defined as any visible atherosclerosis in any of the 18 segments and **any significant stenosis** was defined as any diameter stenosis ≥50%. **Segment involvement score ≥4** (SIS) was defined as four or more coronary segments affected by atherosclerosis.[1] Non-calcified plaque was defined as any non-calcified plaque in the reported segments. Finally, **coronary artery calcium score** (CACS) was dichotomized into ≤100 or >100 Agatston Units, the latter indicating moderate to high risk of future atherosclerotic events.[2]

Additional co-variable description

Data collected from the SCAPIS main questionnaire was used as co-variables in regression modelling. Missing values ranged from zero (sex and age) - 4.2% (financial strain).

Age is given in years.
Systolic blood pressure was measured twice per arm with at least one minute between measurements by automatic Omron Healthcare Co. Ltd, Japan, in both arms in the supine position after 5 minutes rest, with the cuff at heart level. The blood pressure registered was the average of the two systolic blood pressures in the arm with the highest mean blood pressure.
High-density lipoprotein and total cholesterol were analysed using local laboratory standard methods.
The height and weight were measured in centimetres and kg, at the time of the CCTA.
Body mass index was calculated at study visit.
Smoking status was categorised as former or current smoking. Pack years were also calculated.
Alcohol use was considered problematic if the answer to: *How many drinks containing alcohol do you have on a typical day when you are drinking?* Was "5 drinks or more" and to: *How often during the last year have you been unable to remember what happened the night before because you had been drinking?* was "every month or more often".
Participants also reported any **current antihypertensive or lipid-lowering medication** and any **current or past diagnoses**, including myocardial infarction, stroke, or coronary revascularization.
The diagnosis of diabetes was either self-reported or diagnosed at study visit defined as fasting p-glucose ≥7.0 mmol/l day or HbA1c ≥48 mmol/mol.
Family history of myocardial infarction and stroke were separately self-reported.
Level of education was self-reported and higher education, defined as university studies were used in the analyses.
Financial strain was self-reported and defined as answering "yes" to any of the following validated questions: "If you should suddenly find yourself in an situation where you had to find 20,000 kronor in one week, would you manage it?" and "During the last 12 months, have you ever had difficulty in managing the regular expenses for food, rent, bills etc."

sReferences

1. Shaw LJ, Blankstein R, Bax JJ, et al. Society of Cardiovascular Computed Tomography / North American Society of Cardiovascular Imaging - Expert Consensus Document on Coronary CT Imaging of Atherosclerotic Plaque. *J Cardiovasc Comput Tomogr*. Mar-Apr 2021;15(2):93-109.

2. Arnett DK, Blumenthal RS, Albert MA, et al. 2019 ACC/AHA Guideline on the Primary Prevention of Cardiovascular Disease: A Report of the American College of Cardiology/American Heart Association Task Force on Clinical Practice Guidelines. *Circulation*. Sep 10 2019;140(11):e596-e646.

3. Bergstrom G, Persson M, Adiels M, et al. Prevalence of Subclinical Coronary Artery Atherosclerosis in the General Population. *Circulation*. Sep 21 2021;144(12):916-929.

sTables

sTable 1. Sex-specific importance of socioeconomic, lifestyle and conventional risk factors for prediction of any coronary atherosclerosis.

sTable 2. Sex-specific importance of socioeconomic, lifestyle and conventional risk factors for prediction of any significant stenosis ≥50 %.

sTable 3. Sex-specific importance of socioeconomic, lifestyle and conventional risk factors for prediction of SIS ≥4.

sTable 4. Sex-specific importance of socioeconomic, lifestyle and conventional risk factors for prediction of CACS >100.

sTable 5. Sex-specific importance of socioeconomic, lifestyle and conventional risk factors for prediction of any carotid plaque.

Supplementary table 1: Associations between socioeconomic, lifestyle and conventional risk factors and any coronary atherosclerosis in men and women.

	Women (n = 12597)		Men (n=12272)			
**Variable**	**OR (95%CI) (univariate)**	**OR (95%CI) (multivariate)**	**OR (95%CI) (univariate)**	**OR (95%CI) (multivariate)**	**P for interaction (univar)**	**P for interaction (multivar)**
Age	1.11 (1.10-1.12)	1.09 (1.07-1.10)	1.12 (1.11-1.13)	1.10 (1.09-1.11)	0.37	0.94
Ex-smoker	1.75 (1.61-1.91)	1.53 (1.40-1.68)	1.43 (1.32-1.55)	1.14 (1.04-1.24)	**<0.01**	**<0.01**
Smoker	2.43 (2.17-2.74)	1.53 (1.40-1.68)	1.55 (1.38-1.75)	1.39 (1.21-1.59)	-----	-----
Alcohol use (problematic)	1.30 (1.14-1.48)	1.10 (0.95-1.27)	1.20 (1.10-1.30)	1.14 (1.04-1.25)	0.28	0.72
Higher education	0.80 (0.74-0.86)	0.99 (0.90-1.08)	0.86 (0.80-0.93)	1.03 (0.94-1.12)	0.15	0.52
Employed	0.65 (0.59-0.71)	0.99 (0.90-1.08)	0.60 (0.54-0.66)	0.91 (0.80-1.04)	0.23	0.46
Single	1.29 (1.17-1.41)	1.06 (0.96-1.18)	1.16 (0.05-1.28)	1.03 (0.93-1.15)	0.14	0.78
Poor economy	1.14 (1.01-1.29)	1.02 (0.88-1.18)	1.11 (0.97-1.26)	1.02 (0.87-1.19)	0.75	0.95
Foreign born	1.08 (0.98-1.19)	1.02 (0.91-1.15)	1.31 (1.19-1.44)	1.30 (1.15-1.46)	0.006	<0.005
Financial strain	1.08 (0.98-1.19)	1.02 (0.91-1.15)	1.31 (1.19-1.44)	1.30 (1.15-1.46)	**<0.01**	**<0.01**
Sleep apnea	1.42 (1.19-1.70)	1.03 (0.85-1.24)	1.17 (1.05-1.32)	1.01 (0.89-1.15)	0.07	0.88
Stress	0.96 (0.88-1.05)	0.97 (0.88-1.07)	0.95 (0.87-1.06)	1.02 (0.91-1.14)	0.92	0.55
Diabetes	2.27 (1.92-2.68)	1.52 (1.25-1.85)	2.16 (1.88-2.50)	1.41 (1.19-1.66)	0.68	057
Hyperlipidemia	3.05 (2.59-3.59)	2.11 (1.74-2.55)	2.91 (2.49-3.42)	2.13 (1.78-2.55)	0.70	0.97
Hypertension	2.31 (2.10-2.55)	1.60 (1.43-1.79)	2.36 (2.14-2.60)	1.61 (1.43-1.80)	0.78	0.95
Hereditary MI	1.52 (1.32-1.75)	1.44 (1.24-1.68)	1.35 (1.16-1.58)	1.39 (1.17-1.65)	0.27	0.76
BMI high	1.30 (1.19-1.42)	1.01 (0.91-1.11)	1.24 (1.14-1.34)	0.99 (0.89-1.09)	------	-----
BMI obese	1.69 (1.52-1.87)	1.12 (0.97-1.28)	1.75 (1.58-1.94)	1.21 (1.05-1.40)	------	0.34
Waist hip	1.33 (1.28-1.39)	1.08 (1.02-1.13)	1.34 (1.28-1.39)	1.06 (1.01-1.21)	0.87	0.58
MVPA	0.90 (0.87-0.93)	1.00 (0.96-1.04)	0.94 (0.91-0.97)	1.00 (0.97-1.05)	0.12	0.77
Sedentary	1.01 (0.98-1.05)	0.97 (0.93-1.01)	1.00 (0.98-1.03)	0.96 (0.92-1.00)	0.65	0.62
SBP	1.18 (1.15-1.20)	1.08 (1.05-1.11)	1.19 (1.16-1.22)	1.09 (1.06-1.21)	0.43	0.59
LDL	1.23 (1.17-1.27)	1.21 (1.17-1.26)	1.16 (1.12-1.19)	1.25 (1.21-1.30)	**0.01**	0.30
HDL	0.86 (0.83-0.89)	0.97 (0.93-1.01)	0.93 (0.89-0.96)	0.99 (0.95-1.03)	**<0.01**	0.49
CRP	1.15 (1.11-1.19)	1.00 (0.96-1.04)	1.09 (1.06-1.13)	0.97 (0.93-1.01)	**0.03**	0.36

OR = odds ratio, MI = Myocardial Infarction, BMI = Body Mass Index, MVPA = Moderate or Vigorous Physical Activity, SBP = Systolic Blood Pressure, LDL = Low Density Cholesterol, HDL = High Density Cholesterol, CRP = C-Reactive Protein

**Supplementary table 2:** Associations between socioeconomic, lifestyle and conventional risk factors and coronary stenosis ≥50 % in men and women.						
	**Women (n = 12597)**		**Men (n=12272)**			
**Variable**	**OR (95%CI) (univariate)**	**OR (95%CI) (multivariate)**	**OR (95%CI) (univariate)**	**OR (95%CI) (multivariate)**	**P for interaction (univar)**	**P for interaction (multivar)**
Age	1.12 (1.09-1.15)	1.10 (1.06-1.13)	1.11 (1.09-1.13)	1.09(1.07-1.11)	0.61	0.86
Ex-smoker	1.70 (1.30-2.24)	1.50 (1.12-2.01)	1.46 (1.26-1.68)	1.18(1.00-1.38)	**0.02**	**0.02**
Smoker	2.80 (2.02-3.87)	2.34 (1.62-3.37)	1.77 (1.47-2.14)	1.46 (1.17-1.82)	------	------
Alcohol use (problematic)	1.53 (1.07-2.18)	1.38 (0.94-2.01)	1.09 (0.95-1.26)	1.08 (0.92-1.27)	0.08	0.25
Higher education	0.74 (0.58-0.94)	0.95 (0.73-1.25)	0.85 (0.74-0.97)	1.03 (0.89-1.32)	0.33	0.63
Employed	0.70 (0.52-0.91)	1.28 (0.91-1.78)	0.60 (0.52-0.70)	1.08 (0.89-1.20)	0.43	0.41
Single	1.13 (0.84-1.50)	0.86 (0.63-1.17)	1.25 (1.06-1.42)	1.13 (0.94-1.35)	0.53	0.14
Poor economy	1.41 (0.99-2.00)	1.45 (0.98-2.15)	1.17 (0.93-1.47)	1.04 (0.80-1.35)	0.38	0.17
Foreign born	1.12 (0.83-1.52)	1.08 (0.76-1.52)	1.57 (1.35-1.83)	1.50 (1.24-1.8)	0.05	0.10
Sleep apnea	1.14 (0.66-1.97)	0.74 (0.41-1.31)	1.24 (1.02-1.50)	1.05 (0.85-1.29)	0.80	0.25
Stress	0.86 (0.71-1.04)	1.09 (0.82-1.47)	1.08 (0.82-1.41)	0.89 (0.76-1.09)	0.18	0.26
Diabetes	2.74 (1.87-4.02)	1.70 (1.08-2.64)	2.21 (1.84-2.66)	1.63 (1.31-2.03)	0.32	0.91
Hyperlipidemia	3.49 (2.48-4.93)	2.62 81.73-3.96)	2.42 (2.00-2.93)	2.01 (1.60-2.53)	0.07	0.27
Hypertension	2.78 (2.15-3.59)	1.68 (1.25-2.26)	2.14 (1.85-2.47)	1.39 (1.17-1.65)	0.08	0.29
Hereditary MI	1.65 (1.12-2.43)	1.30 (0.85-1.98)	1.83 (1.46-2.30)	1.93 (1.51-2.45)	0.65	0.11
BMI high	1.55 (1.17-2.05)	0.89 (0.68-1.23)	1.37 (1.17-1.62)	0.85 (0.78-1.15)	------	-----
BMI obese	1.89 (1.38-2.59)	0.77 (0.51-1.16)	1.77 (1.46-2.13)	0.85 (0.74-1.23)	------	0.58
Waist hip ratio	1.42 (1.26-1.60)	1.06 (0.92-1.23)	1.48 (1.36-1.61)	1.13 (1.02-1.26)	0.58	0.46
MVPA	0.77 (0.69-0.87)	0.92 (0.81-1.05)	0.92 (0.87-0.98)	1.03 (0.96-1.10)	**<0.01**	0.16
Sedentary	1.09 (0.97-1.21)	0.97 (0.85-1.10)	1.07 (1.01-1.14)	1.03 (0.95-1.10)	0.84	0.41
SBP	1.24 (1.17-1.32)	1.14 (1.07-1.23)	1.23 (1.18-1.27)	1.13 (1.08-1.18)	0.75	0.79
LDL	1.31 (1.18-1.47)	1.33 (1.18-1.51)	1.17 (1.10-1.24)	1.28 (1.20-1.37)	0.06	0.58
HDL	0.73 (0.65-0.81)	0.83 (0.73-0.95)	0.82 (0.76-0.88)	0.91 (0.84-0.99)	0.07	0.28
CRP	1.25 (1.13-1.39)	1.06 (0.94-1.20)	1.16 (1.10-1.23)	1.02 (0.96-1.09)	0.19	0.63
OR = odds ratio, MI = Myocardial Infarction, BMI = Body Mass Index, MVPA = Moderate or Vigorous Physical Activity, SBP = Systolic Blood Pressure, LDL = Low Density Cholesterol, HDL = High Density Cholesterol, CRP = C-Reactive Protein						

**Supplementary table 3:** Associations between socioeconomic, lifestyle and conventional risk factors and SIS ≥4 in men and women.						
	**Women (n = 12597)**		**Men (n=12272)**			
**Variable**	**OR (95%CI) (univariate)**	**OR (95%CI) (multivariate)**	**OR (95%CI) (univariate)**	**OR (95%CI) (multivariate)**	**P for interaction (univar)**	**P for interaction (multivar)**
Age	1.14 (1.12-1.16)	1.01 (1.08-1.13)	1.13 (1.12-1.15)	1.11 (1.10-1.13)	0.78	0.52
Ex-smoker	2-25 (1.86-2.73)	1.91 (1.55-2.36)	1.64 (1.49-1.81)	1.31 (1.17-1.46)	**<0.01**	**<0.01**
Smoker	4.10 (3.29-5.10)	3.45 (2.69-4.50)	1.93 (1.69-2.21)	1.30 (1.17-1.46)	--------	------
Alcohol use (problematic)	1.63 (1.29-2.07)	1.35 (1.04-1.75)	1.21 (1.09-1.33)	1.12 (1.00-1.26)	**0.02**	0.21
Higher education	0.59 (0.50-0.70)	0.86 (0.72-1.04)	0.83 (0.75-0.91)	1.02 (0.91-1.13)	**<0.01**	0.14
Employed	0.55 (0.46-0.66)	1.02 (0.81-1.27)	0.57 (0.51-0.63)	0.93 (0.81-1.07)	0.83	0.50
Single	1.57 (1.31-1.88)	1.23 (1.01-1.51)	1.23 (1.09-1.38)	1.08 (0.94-1.24)	**0.03**	0.28
Poor economy	1.30 (1.02-1.66)	1.10 (0.83-1.47)	1.24 (1.06-1.46)	1.05 (0.86-1.27)	0.74	0.82
Foreign born	1.05 (0.85-1.29)	0.92 (0.72-1.18)	1.38 (1.24-1.55)	1.42 (1.23-1.64)	**0.02**	**<0.01**
Sleep apnea	1.26 (0.89-1.80)	0.78 (0.53-1.16)	1.32 (1.15-1.52)	1.10 (0.94-1.28)	0.82	0.113
Stress	0.87 (0.71-1.05)	0.85 (0.68-1.05)	0.97 (0.86-1.10)	0.99 (0.86-1.14)	0.32	0.23
Diabetes	3.52 (2.75-4.51)	1.86 (1.37-2.53)	2.71 (2.36-3.12	1.66 (1.40-2.00)	0.07	0.54
Hyperlipidemia	4.91 (3.92-6.14)	3.18 (2.40-4.21)	3.38 (2.93-3.89)	2.33 (1.97-2.77)	**<0.01**	0.64
Hypertension	3.19 (2.69-3.79)	1.78 (1.45-2.18)	2.83 (2.55-3.14)	1.79 (1.58-2.03)	0.24	0.96
Hereditary MI	1.88 (1.46-2.42)	1.68 (1.28-2.22)	1.78 (1.50-2.11)	1.39 (1.17-1.65)	0.72	0.40
BMI high	1.37 (1.14-1.66)	0.89 (0.72-1.11	1.38 (1.23-1.55)	0.99 (0.89-1.09)	-----	**0.04**
BMI obese	2.02 (1.65-2.49)	0.94 (0.71-1.25)	2.08 (1.83-2.37)	1.21 (1.05-1.40)	------	-----
Waist hip ratio	1.54 (1.42-1.67)	1.16 (1.05-1.28)	1.42 (1.34-1.51)	1.06 (1.01-1.12)	0.12	0.03
MVPA	0.84 (0.78-0.90)	0.98 (0.90-1.07)	0.89 (0.85-0.92)	1.01 (0.97-1.05)	0.20	0.92
Sedentary	1.03 (0.96-1.12)	0.94 (0.86-1.03)	1.05 (1.00-1.09)	0.96 (0.92-1.00)	0.77	0.48
SBP	1.24 (1.19-1.30)	1.13 (1.08-1.19)	1.25 (1.21-1.28)	1.09 (1.06-1.12)	0.92	0.97
LDL	1.20 (1.11-1.28)	1.28 (1.17-1.39)	1.09 (1.05-1.14)	1.25 (1.21-1.30)	**0.03**	0.68
HDL	0.80 (0.74-0.86)	0.95 (0.87-1.04)	0.90 (0.86-0.95)	0.99 (0.95-1.03)	**<0.01**	0.44
CRP	1.22 (1.14-1.30)	1.00 (0.92-1.09)	1.13 (1.09-1.17)	0.97 (0.93-1.01)	0.05	0.65
OR = odds ratio, MI = Myocardial Infarction, BMI = Body Mass Index, MVPA = Moderate or Vigorous Physical Activity, SBP = Systolic Blood Pressure, LDL = Low Density Cholesterol, HDL = High Density Cholesterol, CRP = C-Reactive Protein						

s

Supplementary table 4: Associations between socioeconomic, lifestyle and conventional risk factors and CACS >100 in men and women.

	Women (n = 12597)		Men (n=12272)			
**Variable**	**OR (95%CI) (univariate)**	**OR (95%CI) (multivariate)**	**OR (95%CI) (univariate)**	**OR (95%CI) (multivariate)**	**P for interaction (univar)**	**P for interaction (multivar)**
Age	1.14 (1.12-1.16)	1.11 (1.08-1.13)	1.15 (1.14-1.17)	1.13 (1.12-1.15)	0.37	0.11
Ex-smoker	2.15 (1.79-2.57)	1.75 (1.44-2.13)	1.63 (1.47-1.81)	1.25 (1.11-1.40)	**<0.01**	**<0.01**
Smoker	3.59 (2.90-4.50)	3.12 (2.45-3.99)	1.69 (1.47-1.95)	1.49 (1.26-1.76)	--------	-------
Alcohol use (problematic)	1.45 (1.15-1.83)	1.12 (0.87-1.46)	1.25 (1.13-1.38)	1.20 (1.07-1.35)	0.25	0.63
Higher education	0.68 (0.58-0.79)	0.93 (0.78-1.11)	0.85 (0.77-0.94)	1.03 (0.92-1.15)	**0.01**	0.37
Employed	0.57 (0.48-0.68)	1.02 (0.82-1.26)	0.56 (0.50-0.63)	0.93 (0.80-1.08)	0.90	0.49
Single	1.59 (1.34-1.89)	1.28 (1.06-1.55)	1.20 (1.06-1.36)	1.06 (0.92-1.22)	**<0.01**	0.12
Poor economy	1.38 (1.10-1.74)	1.23 (0.94-1.62	1.13 (0.95-1.33)	1.01 (0.82-1.23)	0.15	0.24
Foreign born	1.09 (0.90-1.33)	1.02 (0.81-1.28)	1.25 (1.11-1.41)	1.26 (1.09-1.46)	0.25	0.13
Sleep apnea	1.49 (1.08-2.04	0.97 (0.69-1.38)	1.30 (1.13-1.50)	1.10 (0.94-1.29)	0.46	0.51
Stress	0.87 (0.73-1.05)	0.88 (0.72-1.08)	0.93 (0.81-1.06)	0.99 (0.85-1.14)	0.62	0.39
Diabetes	2.98 (2.32-3.83)	1.76 (1.30-2.39)	2.56 (2.22-2.96)	1.56 (1.31-1.85)	0.31	0.51
Hyperlipidemia	4.18 (3.37-5.19)	2.46 (1.85-3.25)	3.35 (2.92-3.84)	2.04 (1.71-2.44)	0.17	0.27
Hypertension	2.71 (2.29-3.21)	1.60 (1.31-1.95)	2.74 (2.46-3.04)	1.71 (1.50-1.94)	0.93	0.55
Hereditary MI	1.76 (1.38-2.26)	1.55 (1.18-2.03)	1.68 (1.39-1.99)	1.76 (1.45-2.14)	0.71	0.45
BMI high	1.28 (1.07-1.53)	0.88 (0.72-1.08)	1.28 (1.14-1.44)	1.01 (0.88-1.17)	-----	0.18
BMI obese	1.71 (1.40-2.09)	0.92 (0.70-1.20)	1.92 (1.68-2.20)	1.32 (1.10-1.59)	-----	------
Waist hip	1.45 (1.34-1.57)	1.16 (1.05-1.28)	1.42 (1.34-1.50)	1.03 (0.95-1.11)	0.60	0.05
MVPA	0.86 (0.80-0.92)	0.99 (0.91-1.08)	0.92 (0.88-0.95)	1.00 (0.95-1.05)	0.14	0.82
Sedentary	1.04 (0.97-1.12)	0.99 (0.91-1.08)	1.00 (0.96-1.05)	0.94 (0.90-1.00)	0.35	0.34
SBP	1.24 (1.19-1.29)	1.14 (1.09-1.19)	1.22 (1.18-1.25)	1.09 (1.05-1.13)	0.47	0.15
LDL	1.15 (1.08-1.24)	1.21 (1.11-1.30)	1.03 (0.99-1.08)	1.17 (1.11-1.23)	**0.01**	0.46
HDL	0.88 (0.82-0.95)	1.05 (0.97-1.15)	0.94 (0.89-0.98)	0.99 (0.93-1.05)	0.19	0.25
CRP	1.18 (1.10-1.25)	1.01 (0.93-1.10)	1.11 (1.07-1.16)	0.97 (0.92-1.01)	0.14	0.37

OR = odds ratio, MI = Myocardial Infarction, BMI = Body Mass Index, MVPA = Moderate or Vigorous Physical Activity, SBP = Systolic Blood Pressure, LDL = Low Density Cholesterol, HDL = High Density Cholesterol, CRP = C-Reactive Protein

**Supplementary table 5:** Associations between socioeconomic, lifestyle and conventional risk factors and any carotid plaque in men and women.						
	**Women (n = 12597)**		**Men (n=12272)**			
**Variable**	**OR (95%CI) (univariate)**	**OR (95%CI) (multivariate)**	**OR (95%CI) (univariate)**	**OR (95%CI) (multivariate)**	**P for interaction (univar)**	**P for interaction (multivar)**
Age	1.08 (1.07-1.09)	1.05 (1.04-1.06)	1.09 (1.08-1.09)	1.07 (1.06-1.08)	0.23	0.98
Ex-smoker	1.39 (1.29-1.50)	1.30 (1.20-1.42)	1.32 (1.22-1.43)	1.17 (1.07-1.28)	**0.03**	1.13
Smoker	1.89 (1.69-2.12)	1.88 (1.66-2.13)	1.57 (1.40-1.77)	1.47 (1.28-1.68)	-----	-----
Alcohol use (problematic)	1.02 (0.91-1.16)	0.92 (0.81-1.05)	1.13 (1.04-1.22)	1.08 (0.98-1.18)	0.21	0.86
Higher education	0.89 (0.83-0.95)	1.00 (0.92-1.08)	0.85 (0.79-0.91)	0.97 (0.89-1.05)	0.42	1.04
Employed	0.76 (0.70-084)	1.04 (0.94-1.17)	0.74 (0.67-0.82)	0.97 (0.89-1.05)	0.71	1.01
Single	1.16 (1.06-1.26)	1.02 (0.93-1.12)	1.12 (1.01-1.24)	1.02 (0.91-1.17)	0.63	1.00
Poor economy	1.10 (0.98-1.24)	1.11 (0.97-1.27)	1.10 (0.96-1.26)	1.06 (0.91-1.25)	0.96	1.05
Foreign born	1.05 (0.95-1.15)	1.05 (0.95-1.17)	1.11 (1.01-1.22)	1.08 (0.96-1.21)	0.39	1.02
Sleep apnea	0.98 (0.83-1.16)	0.82 (0.69-0.99)	1.12 (0.99-1.26)	1.05 (0.93-1.20)	0.20	0.78
Stress	1.03 (0.95-1.12)	1.10 (1.01-1.20)	0.94 (0.85-1.03)	0.98 (0.87-1.09)	0.15	1.13
Diabetes	1.38 (1.17-1.63)	1.18 (0.98-1.43)	1.47 (1.28-1.69)	1.22 (1.04-1.43)	0.56	0.97
Hyperlipidemia	2.05 (1.75-2.41)	1.76 (1.45-2.13)	1.82 (1.58-2.08)	1.69 (1.42-2.01)	0.53	1.04
Hypertension	1.70 (1.54-1.87)	1.34 (1.20-1.49)	1.62 (1.47-1.79)	1.25 (1.11-1.40)	0.52	1.07
Hereditary MI	1.17 (1.02-1.39)	1.10 (0.95-1.27)	1.01 (0.86-1.18)	1.01 (0.86-1.20)	0.16	1.09
BMI high	1.08 (1.00-1.17)	0.87 (0.79-0.95)	1.11 (1.02-1.21)	0.91 (0.82-1.01)	-----	0.98
BMI obese	1.03 (0.94-1.14)	0.74 (0.65-0.84)	1.15 (1.04-1.28)	0.81 (0.70-0.93)	-----	-----
Waist hip	1.10 (1.06-1.14)	0.95 (0.91-1.00)	1.14 (1.09-1.19)	0.93 (0.88-0.98)	0.20	1.02
MVPA	0.96 (0.93-0.99)	1.02 (0.98-1.06)	0.96 (0.93-0.99)	0.98 (0.95-1.02)	0.88	1.04
Sedentary	0.98 (0.95-1.02)	0.98 (0.94-1.02)	0.96 (0.92-0.99)	0.92 (0.89-0.96)	0.22	1.06
SBP	1.17 (1.15-1.20)	1.13 (1.11-1.16)	1.22 (1.19-1.25)	1.19 (1.15-1.22)	**0.01**	0.96
LDL	1.24 (1.21-1.28)	1.24 (1.19-1.28)	1.21 (1.17-1.25)	1.26 (1.21-1.31)	0.21	0.98
HDL	0.92 (0.89-0.95)	0.94 (0.91-1.04)	0.93 (0.89-0.96)	0.92 (0.88-0.96)	0.80	1.02
CRP	1.05 (1.02-1.08)	1.00 (0.97-1.04)	1.08 (1.05-1.12)	1.05 (1.04-1.06)	0.19	0.98
OR = odds ratio, MI = Myocardial Infarction, BMI = Body Mass Index, MVPA = Moderate or Vigorous Physical Activity, SBP = Systolic Blood Pressure, LDL = Low Density Cholesterol, HDL = High Density Cholesterol, CRP = C-Reactive Protein						
